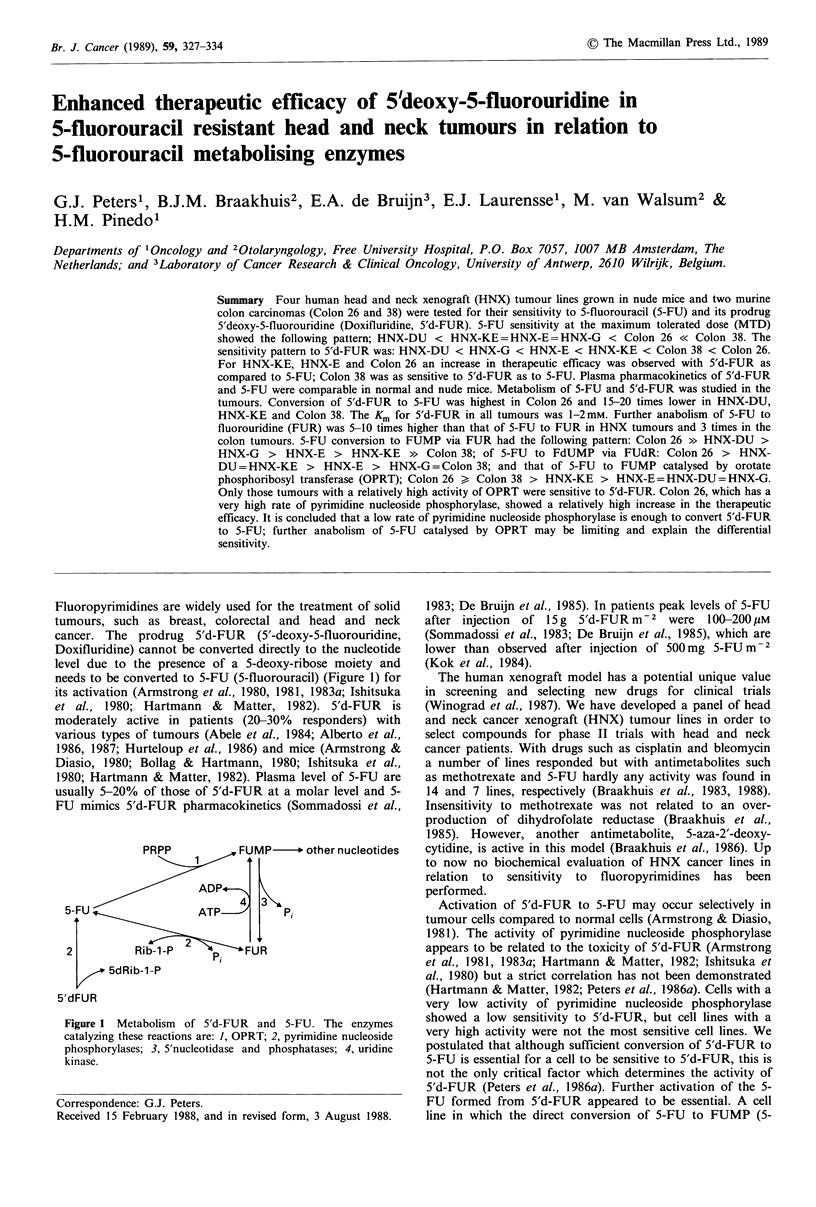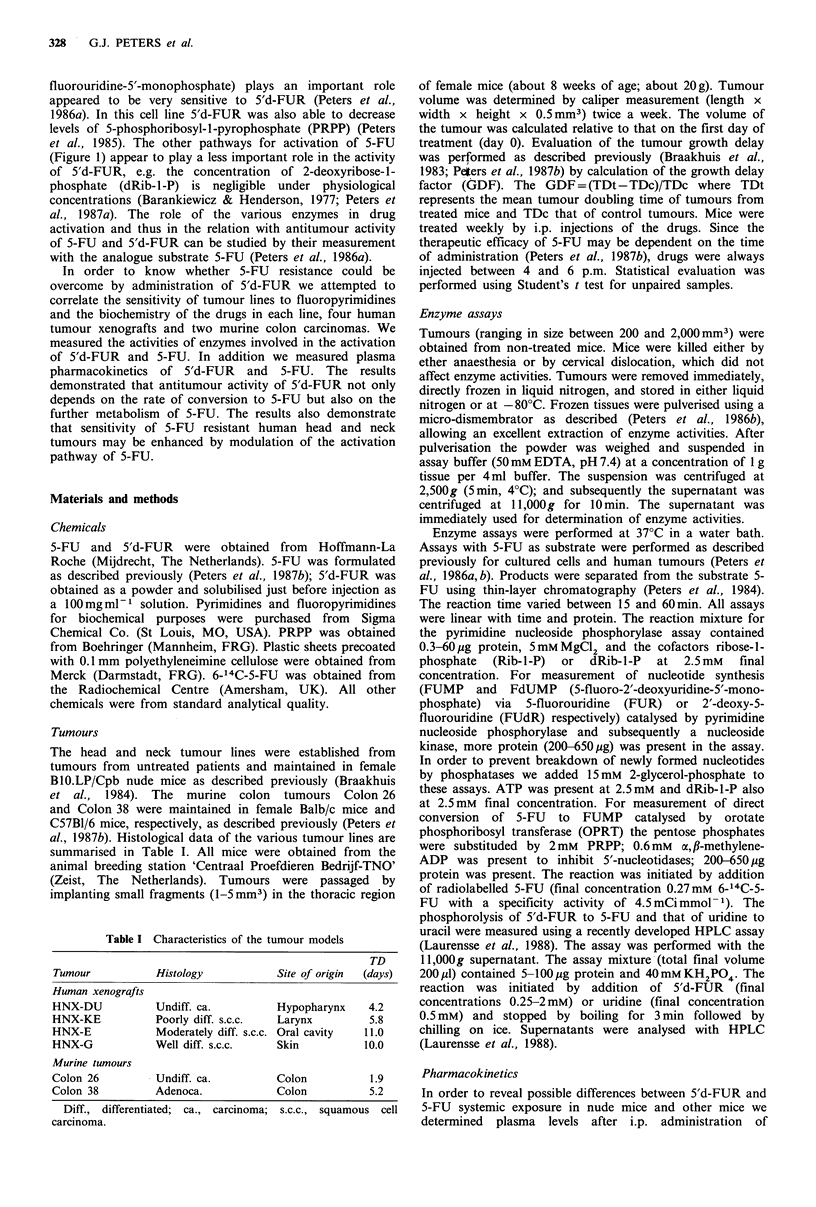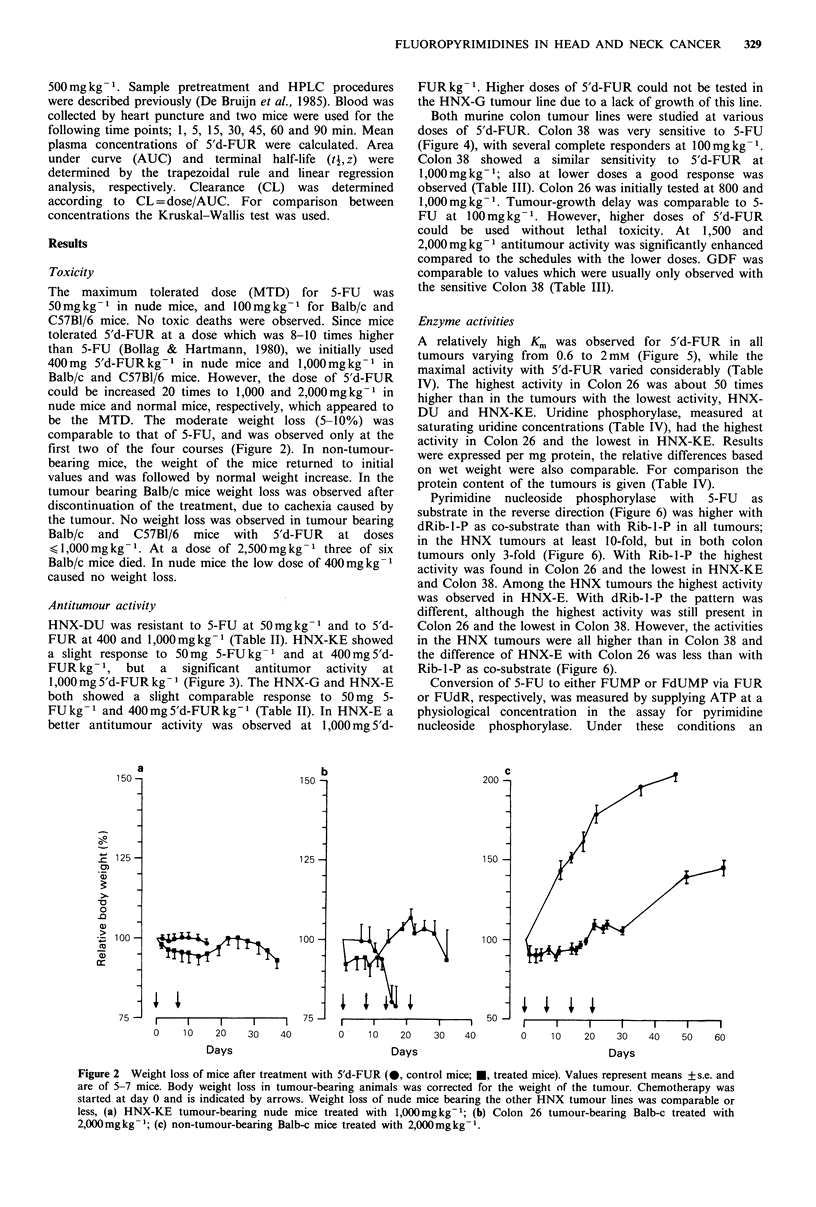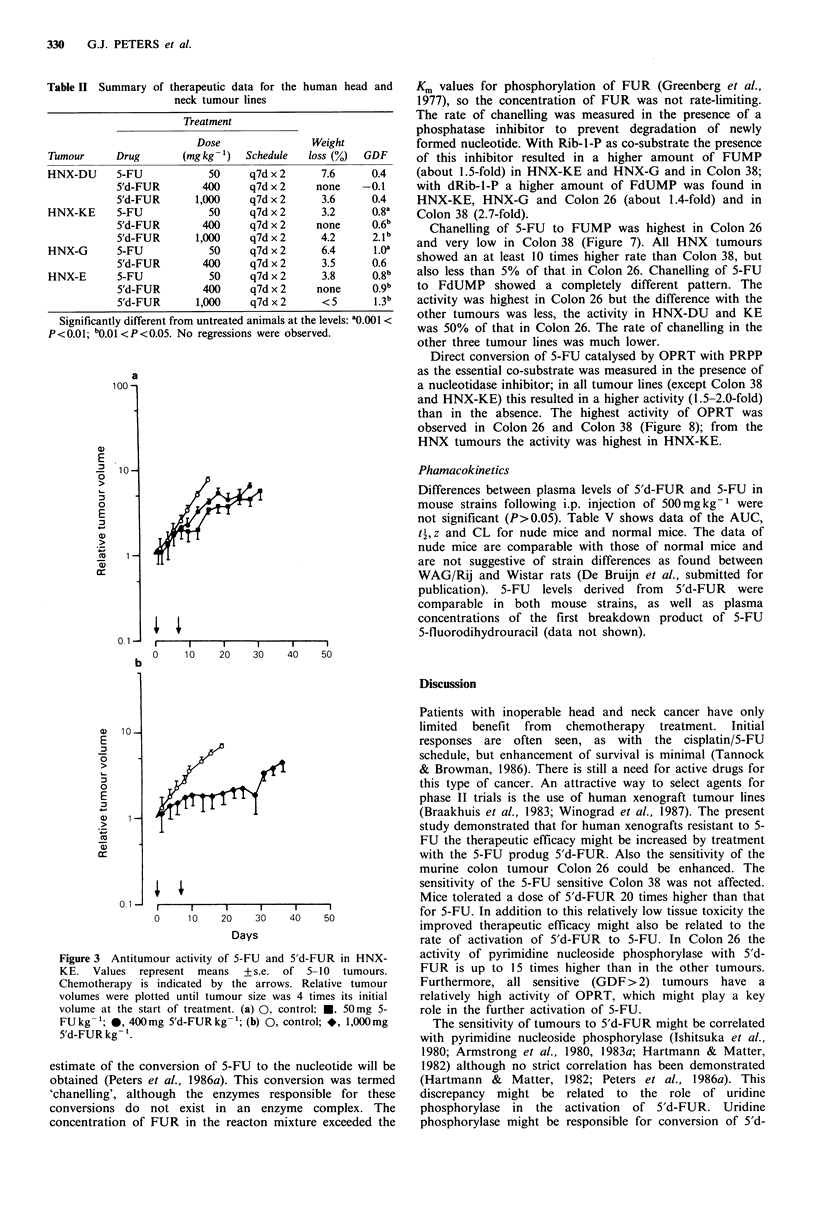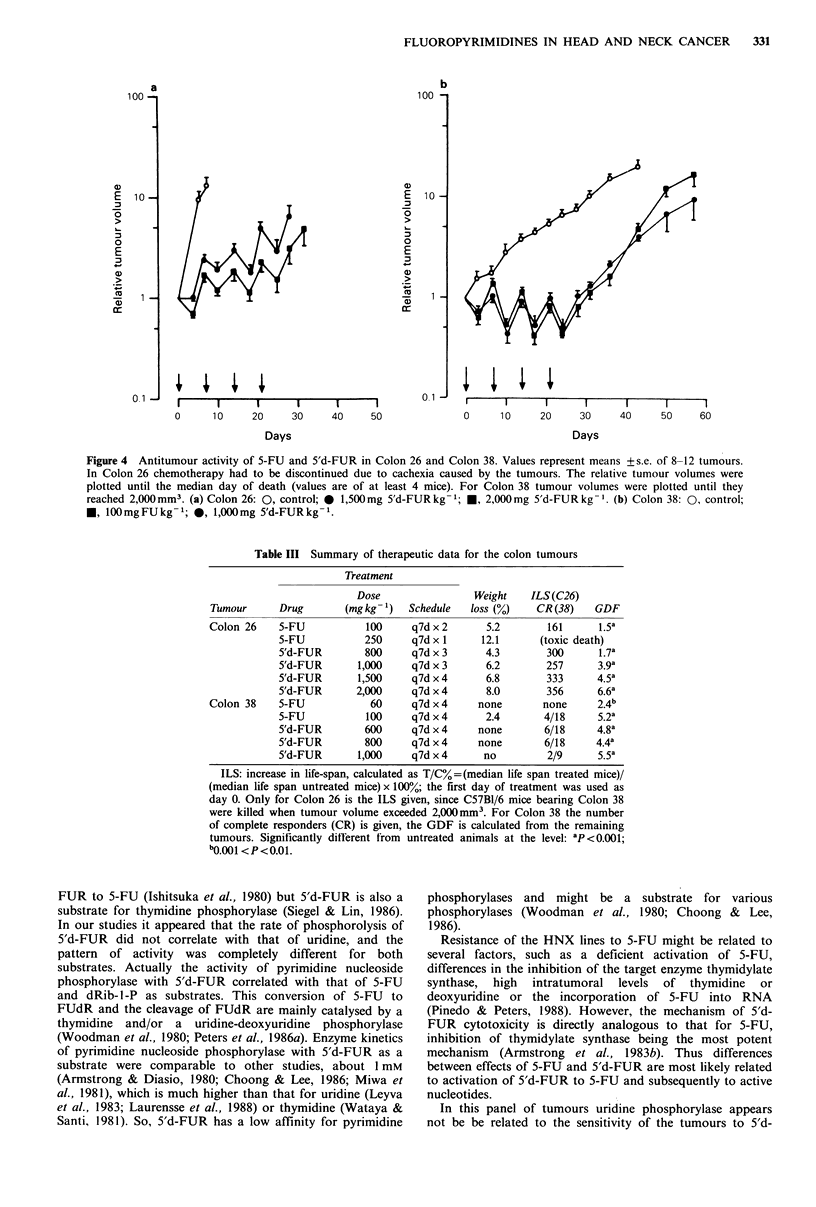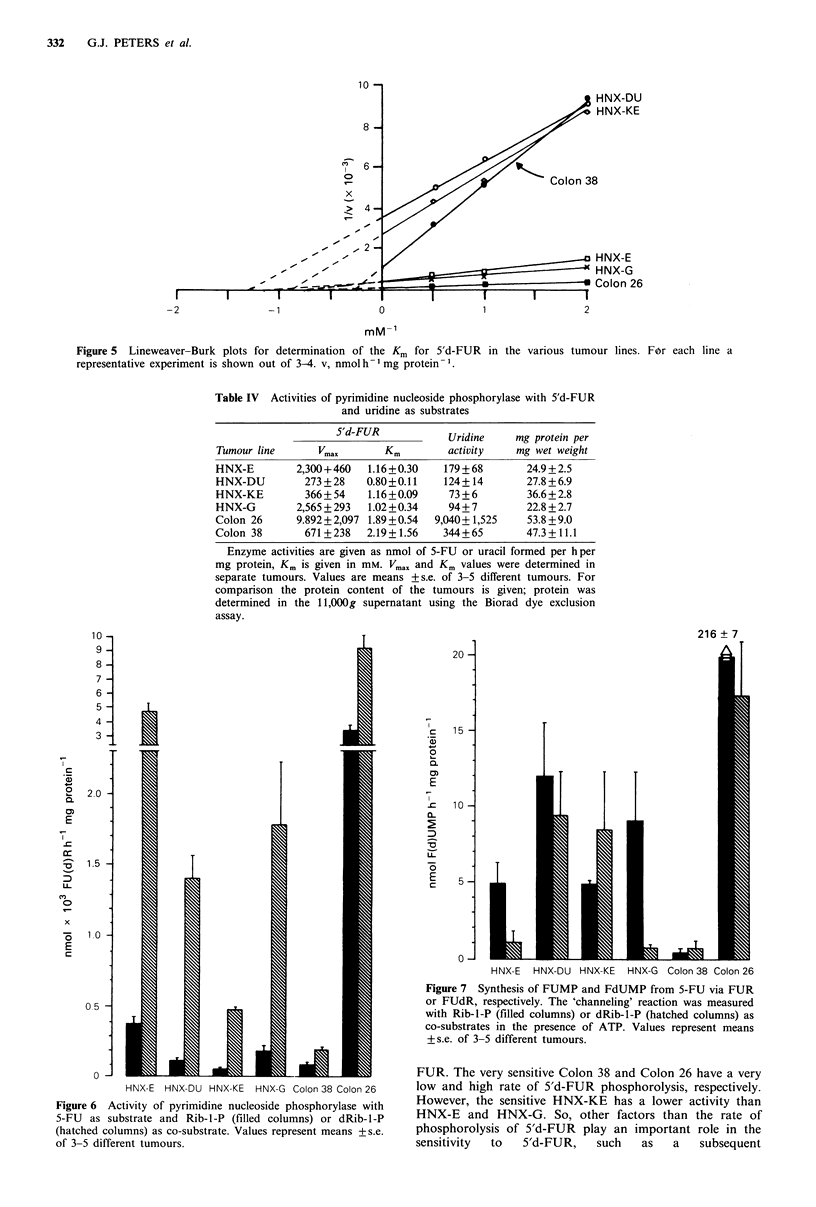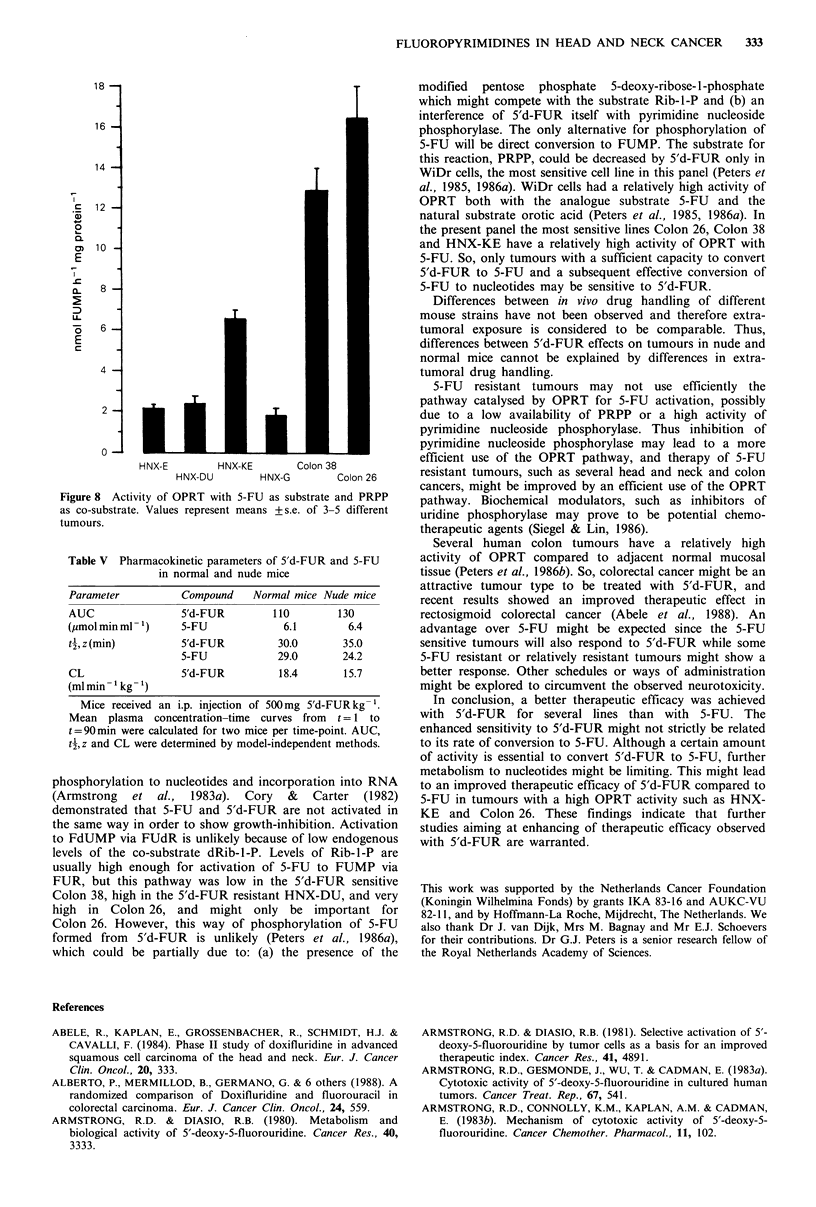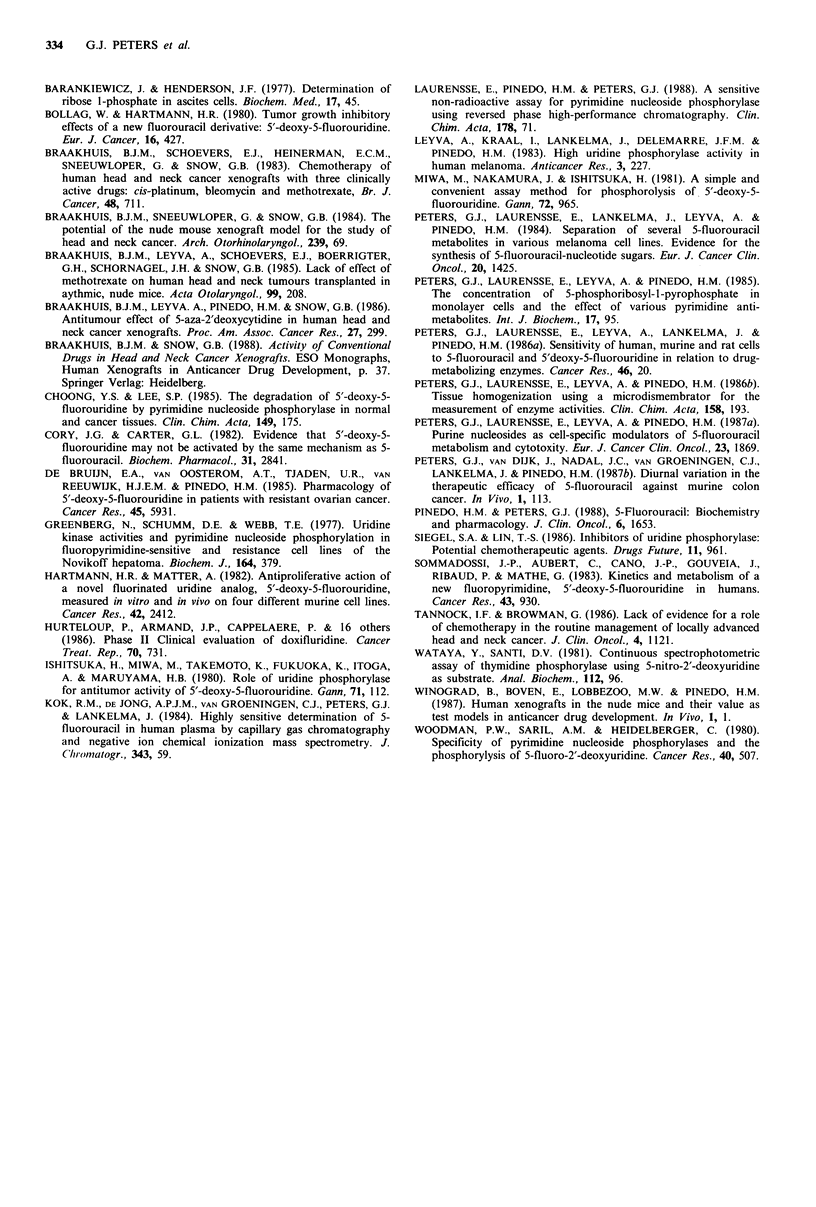# Enhanced therapeutic efficacy of 5'deoxy-5-fluorouridine in 5-fluorouracil resistant head and neck tumours in relation to 5-fluorouracil metabolising enzymes.

**DOI:** 10.1038/bjc.1989.65

**Published:** 1989-03

**Authors:** G. J. Peters, B. J. Braakhuis, E. A. de Bruijn, E. J. Laurensse, M. van Walsum, H. M. Pinedo

**Affiliations:** Department of Oncology, Free University Hospital, Amsterdam, The Netherlands.

## Abstract

Four human head and neck xenograft (HNX) tumour lines grown in nude mice and two murine colon carcinomas (Colon 26 and 38) were tested for their sensitivity to 5-fluorouracil (5-FU) and its prodrug 5'deoxy-5-fluorouridine (Doxifluridine, 5'd-FUR). 5-FU sensitivity at the maximum tolerated dose (MTD) showed the following pattern; HNX-DU less than HNX-KE = HNX-E = HNX-G less than Colon 26 much less than Colon 38. The sensitivity pattern to 5'd-FUR was: HNX-DU less than HNX-G less than HNX-E less than HNX-KE less than Colon 38 less than Colon 26. For HNX-KE, HNX-E and Colon 26 an increase in therapeutic efficacy was observed with 5'd-FUR as compared to 5-FU; Colon 38 was as sensitive to 5'd-FUR as to 5-FU. Plasma pharmacokinetics of 5'd-FUR and 5-FU were comparable in normal and nude mice. Metabolism of 5-FU and 5'd-FUR was studied in the tumours. Conversion of 5'd-FUR to 5-FU was highest in Colon 26 and 15-20 times lower in HNX-DU, HNX-KE and Colon 38. The Km for 5'd-FUR in all tumours was 1-2 mM. Further anabolism of 5-FU to fluorouridine (FUR) was 5-10 times higher than that of 5-FU to FUR in HNX tumours and 3 times in the colon tumours. 5-FU conversion to FUMP via FUR had the following pattern: Colon 26 much greater than HNX-DU greater than HNX-G greater than HNX-E greater than HNX-KE much greater than Colon 38; of 5-FU to FdUMP via FUdR: Colon 26 greater than HNX-DU = HNX-KE greater than HNX-E greater than HNX-G = Colon 38; and that of 5-FU to FUMP catalysed by orotate phosphoribosyl transferase (OPRT); Colon 26 greater than or equal to Colon 38 greater than HNX-KE greater than HNX-E = HNX-DU = HNX-G. Only those tumours with a relatively high activity of OPRT were sensitive to 5'd-FUR. Colon 26, which has a very high rate of pyrimidine nucleoside phosphorylase, showed a relatively high increase in the therapeutic efficacy. It is concluded that a low rate of pyrimidine nucleoside phosphorylase is enough to convert 5'd-FUR to 5-FU; further anabolism of 5-FU catalysed by OPRT may be limiting and explain the differential sensitivity.


*Br. J. Cancer* (1989), **59**, 327–334

© The Macmillan Press Ltd., 1989

### Enhanced therapeutic efficacy of 5′deoxy-5-fluorouridine in 5-fluorouracil resistant head and neck tumours in relation to 5-fluorouracil metabolising enzymes

G.J. Peters[1], B.J.M. Braakhuis[2], E.A. de Bruijn[3], E.J. Laurensse[1], M. van Walsum[2] & H.M. Pinedo[1]

*Departments of [1]Oncology and [2]Otolaryngology, Free University Hospital, P.O. Box 7057, 1007 MB Amsterdam, The Netherlands; and [3]Laboratory of Cancer Research & Clinical Oncology, University of Antwerp, 2610 Wilrijk, Belgium.*

**Summary** Four human head and neck xenograft (HNX) tumour lines grown in nude mice and two murine colon carcinomas (Colon 26 and 38) were tested for their sensitivity to 5-fluorouracil (5-FU) and its prodrug 5′deoxy-5-fluorouridine (Doxifluridine, 5′d-FUR). 5-FU sensitivity at the maximum tolerated dose (MTD) showed the following pattern; HNX-DU < HNX-KE = HNX-E = HNX-G < Colon 26 ≪ Colon 38. The sensitivity pattern to 5′d-FUR was: HNX-DU < HNX-G < HNX-E < HNX-KE < Colon 38 < Colon 26. For HNX-KE, HNX-E and Colon 26 an increase in therapeutic efficacy was observed with 5′d-FUR as compared to 5-FU; Colon 38 was as sensitive to 5′d-FUR as to 5-FU. Plasma pharmacokinetics of 5′d-FUR and 5-FU were comparable in normal and nude mice. Metabolism of 5-FU and 5′d-FUR was studied in the tumours. Conversion of 5′d-FUR to 5-FU was highest in Colon 26 and 15–20 times lower in HNX-DU, HNX-KE and Colon 38. The $K_m$ for 5′d-FUR in all tumours was 1–2 mM. Further anabolism of 5-FU to fluorouridine (FUR) was 5–10 times higher than that of 5-FU to FUR in HNX tumours and 3 times in the colon tumours. 5-FU conversion to FUMP via FUR had the following pattern: Colon 26 ≫ HNX-DU > HNX-G > HNX-E > HNX-KE ≫ Colon 38; of 5-FU to FdUMP via FUdR: Colon 26 > HNX-DU = HNX-KE > HNX-E > HNX-G = Colon 38; and that of 5-FU to FUMP catalysed by orotate phosphoribosyl transferase (OPRT); Colon 26 ⩾ Colon 38 > HNX-KE > HNX-E = HNX-DU = HNX-G. Only those tumours with a relatively high activity of OPRT were sensitive to 5′d-FUR. Colon 26, which has a very high rate of pyrimidine nucleoside phosphorylase, showed a relatively high increase in the therapeutic efficacy. It is concluded that a low rate of pyrimidine nucleoside phosphorylase is enough to convert 5′d-FUR to 5-FU; further anabolism of 5-FU catalysed by OPRT may be limiting and explain the differential sensitivity.

Fluoropyrimidines are widely used for the treatment of solid tumours, such as breast, colorectal and head and neck cancer. The prodrug 5′d-FUR (5′-deoxy-5-fluorouridine, Doxifluridine) cannot be converted directly to the nucleotide level due to the presence of a 5-deoxy-ribose moiety and needs to be converted to 5-FU (5-fluorouracil) (Figure 1) for its activation (Armstrong *et al.*, 1980, 1981, 1983*a*; Ishitsuka *et al.*, 1980; Hartmann & Matter, 1982). 5′d-FUR is moderately active in patients (20–30% responders) with various types of tumours (Abele *et al.*, 1984; Alberto *et al.*, 1986, 1987; Hurteloup *et al.*, 1986) and mice (Armstrong & Diasio, 1980; Bollag & Hartmann, 1980; Ishitsuka *et al.*, 1980; Hartmann & Matter, 1982). Plasma level of 5-FU are usually 5–20% of those of 5′d-FUR at a molar level and 5-FU mimics 5′d-FUR pharmacokinetics (Sommadossi *et al.*, 1983; De Bruijn *et al.*, 1985). In patients peak levels of 5-FU after injection of 15 g 5′d-FUR $m^{-2}$ were 100–200 $\mu$M (Sommadossi *et al.*, 1983; De Bruijn *et al.*, 1985), which are lower than observed after injection of 500 mg 5-FU $m^{-2}$ (Kok *et al.*, 1984).

**Figure 1** Metabolism of 5′d-FUR and 5-FU. The enzymes catalyzing these reactions are: *1*, OPRT; *2*, pyrimidine nucleoside phosphorylases; *3*, 5′nucleotidase and phosphatases; *4*, uridine kinase.

The human xenograft model has a potential unique value in screening and selecting new drugs for clinical trials (Winograd *et al.*, 1987). We have developed a panel of head and neck cancer xenograft (HNX) tumour lines in order to select compounds for phase II trials with head and neck cancer patients. With drugs such as cisplatin and bleomycin a number of lines responded but with antimetabolites such as methotrexate and 5-FU hardly any activity was found in 14 and 7 lines, respectively (Braakhuis *et al.*, 1983, 1988). Insensitivity to methotrexate was not related to an overproduction of dihydrofolate reductase (Braakhuis *et al.*, 1985). However, another antimetabolite, 5-aza-2′-deoxycytidine, is active in this model (Braakhuis *et al.*, 1986). Up to now no biochemical evaluation of HNX cancer lines in relation to sensitivity to fluoropyrimidines has been performed.

Activation of 5′d-FUR to 5-FU may occur selectively in tumour cells compared to normal cells (Armstrong & Diasio, 1981). The activity of pyrimidine nucleoside phosphorylase appears to be related to the toxicity of 5′d-FUR (Armstrong *et al.*, 1981, 1983*a*; Hartmann & Matter, 1982; Ishitsuka *et al.*, 1980) but a strict correlation has not been demonstrated (Hartmann & Matter, 1982; Peters *et al.*, 1986*a*). Cells with a very low activity of pyrimidine nucleoside phosphorylase showed a low sensitivity to 5′d-FUR, but cell lines with a very high activity were not the most sensitive cell lines. We postulated that although sufficient conversion of 5′d-FUR to 5-FU is essential for a cell to be sensitive to 5′d-FUR, this is not the only critical factor which determines the activity of 5′d-FUR (Peters *et al.*, 1986*a*). Further activation of the 5-FU formed from 5′d-FUR appeared to be essential. A cell line in which the direct conversion of 5-FU to FUMP (5-

Correspondence: G.J. Peters.
Received 15 February 1988, and in revised form, 3 August 1988.

fluorouridine-5′-monophosphate) plays an important role appeared to be very sensitive to 5′d-FUR (Peters *et al.*, 1986*a*). In this cell line 5′d-FUR was also able to decrease levels of 5-phosphoribosyl-1-pyrophosphate (PRPP) (Peters *et al.*, 1985). The other pathways for activation of 5-FU (Figure 1) appear to play a less important role in the activity of 5′d-FUR, e.g. the concentration of 2-deoxyribose-1-phosphate (dRib-1-P) is negligible under physiological concentrations (Barankiewicz & Henderson, 1977; Peters *et al.*, 1987*a*). The role of the various enzymes in drug activation and thus in the relation with antitumour activity of 5-FU and 5′d-FUR can be studied by their measurement with the analogue substrate 5-FU (Peters *et al.*, 1986*a*).

In order to know whether 5-FU resistance could be overcome by administration of 5′d-FUR we attempted to correlate the sensitivity of tumour lines to fluoropyrimidines and the biochemistry of the drugs in each line, four human tumour xenografts and two murine colon carcinomas. We measured the activities of enzymes involved in the activation of 5′d-FUR and 5-FU. In addition we measured plasma pharmacokinetics of 5′d-FUR and 5-FU. The results demonstrated that antitumour activity of 5′d-FUR not only depends on the rate of conversion to 5-FU but also on the further metabolism of 5-FU. The results also demonstrate that sensitivity of 5-FU resistant human head and neck tumours may be enhanced by modulation of the activation pathway of 5-FU.

### Materials and methods

### *Chemicals*

5-FU and 5′d-FUR were obtained from Hoffmann-La Roche (Mijdrecht, The Netherlands). 5-FU was formulated as described previously (Peters *et al.*, 1987*b*); 5′d-FUR was obtained as a powder and solubilised just before injection as a $100\,mg\,ml^{-1}$ solution. Pyrimidines and fluoropyrimidines for biochemical purposes were purchased from Sigma Chemical Co. (St Louis, MO, USA). PRPP was obtained from Boehringer (Mannheim, FRG). Plastic sheets precoated with 0.1 mm polyethyleneimine cellulose were obtained from Merck (Darmstadt, FRG). 6-$^{14}$C-5-FU was obtained from the Radiochemical Centre (Amersham, UK). All other chemicals were from standard analytical quality.

### *Tumours*

The head and neck tumour lines were established from tumours from untreated patients and maintained in female B10.LP/Cpb nude mice as described previously (Braakhuis *et al.*, 1984). The murine colon tumours Colon 26 and Colon 38 were maintained in female Balb/c mice and C57Bl/6 mice, respectively, as described previously (Peters *et al.*, 1987*b*). Histological data of the various tumour lines are summarised in Table I. All mice were obtained from the animal breeding station 'Centraal Proefdieren Bedrijf-TNO' (Zeist, The Netherlands). Tumours were passaged by implanting small fragments ($1-5\,mm^3$) in the thoracic region of female mice (about 8 weeks of age; about 20 g). Tumour volume was determined by caliper measurement (length × width × height × $0.5\,mm^3$) twice a week. The volume of the tumour was calculated relative to that on the first day of treatment (day 0). Evaluation of the tumour growth delay was performed as described previously (Braakhuis *et al.*, 1983; Peters *et al.*, 1987*b*) by calculation of the growth delay factor (GDF). The GDF = (TDt − TDc)/TDc where TDt represents the mean tumour doubling time of tumours from treated mice and TDc that of control tumours. Mice were treated weekly by i.p. injections of the drugs. Since the therapeutic efficacy of 5-FU may be dependent on the time of administration (Peters *et al.*, 1987*b*), drugs were always injected between 4 and 6 p.m. Statistical evaluation was performed using Student's *t* test for unpaired samples.

**Table I** Characteristics of the tumour models

| *Tumour* | *Histology* | *Site of origin* | *TD (days)* |
|---|---|---|---|
| *Human xenografts* | | | |
| HNX-DU | Undiff. ca. | Hypopharynx | 4.2 |
| HNX-KE | Poorly diff. s.c.c. | Larynx | 5.8 |
| HNX-E | Moderately diff. s.c.c. | Oral cavity | 11.0 |
| HNX-G | Well diff. s.c.c. | Skin | 10.0 |
| *Murine tumours* | | | |
| Colon 26 | Undiff. ca. | Colon | 1.9 |
| Colon 38 | Adenoca. | Colon | 5.2 |

Diff., differentiated; ca., carcinoma; s.c.c., squamous cell carcinoma.

### *Enzyme assays*

Tumours (ranging in size between 200 and $2{,}000\,mm^3$) were obtained from non-treated mice. Mice were killed either by ether anaesthesia or by cervical dislocation, which did not affect enzyme activities. Tumours were removed immediately, directly frozen in liquid nitrogen, and stored in either liquid nitrogen or at −80°C. Frozen tissues were pulverised using a micro-dismembrator as described (Peters *et al.*, 1986*b*), allowing an excellent extraction of enzyme activities. After pulverisation the powder was weighed and suspended in assay buffer (50 mM EDTA, pH 7.4) at a concentration of 1 g tissue per 4 ml buffer. The suspension was centrifuged at 2,500 ***g*** (5 min, 4°C); and subsequently the supernatant was centrifuged at 11,000 ***g*** for 10 min. The supernatant was immediately used for determination of enzyme activities.

Enzyme assays were performed at 37°C in a water bath. Assays with 5-FU as substrate were performed as described previously for cultured cells and human tumours (Peters *et al.*, 1986*a*,*b*). Products were separated from the substrate 5-FU using thin-layer chromatography (Peters *et al.*, 1984). The reaction time varied between 15 and 60 min. All assays were linear with time and protein. The reaction mixture for the pyrimidine nucleoside phosphorylase assay contained 0.3–60 $\mu$g protein, 5 mM $MgCl_2$ and the cofactors ribose-1-phosphate (Rib-1-P) or dRib-1-P at 2.5 mM final concentration. For measurement of nucleotide synthesis (FUMP and FdUMP (5-fluoro-2′-deoxyuridine-5′-monophosphate) via 5-fluorouridine (FUR) or 2′-deoxy-5-fluorouridine (FUdR) respectively) catalysed by pyrimidine nucleoside phosphorylase and subsequently a nucleoside kinase, more protein (200–650 $\mu$g) was present in the assay. In order to prevent breakdown of newly formed nucleotides by phosphatases we added 15 mM 2-glycerol-phosphate to these assays. ATP was present at 2.5 mM and dRib-1-P also at 2.5 mM final concentration. For measurement of direct conversion of 5-FU to FUMP catalysed by orotate phosphoribosyl transferase (OPRT) the pentose phosphates were substituted by 2 mM PRPP; 0.6 mM $\alpha,\beta$-methylene-ADP was present to inhibit 5′-nucleotidases; 200–650 $\mu$g protein was present. The reaction was initiated by addition of radiolabelled 5-FU (final concentration 0.27 mM 6-$^{14}$C-5-FU with a specificity activity of $4.5\,mCi\,mmol^{-1}$). The phosphorolysis of 5′d-FUR to 5-FU and that of uridine to uracil were measured using a recently developed HPLC assay (Laurensse *et al.*, 1988). The assay was performed with the 11,000 ***g*** supernatant. The assay mixture (total final volume 200 $\mu$l) contained 5–100 $\mu$g protein and 40 mM $KH_2PO_4$. The reaction was initiated by addition of 5′d-FUR (final concentrations 0.25–2 mM) or uridine (final concentration 0.5 mM) and stopped by boiling for 3 min followed by chilling on ice. Supernatants were analysed with HPLC (Laurensse *et al.*, 1988).

### *Pharmacokinetics*

In order to reveal possible differences between 5′d-FUR and 5-FU systemic exposure in nude mice and other mice we determined plasma levels after i.p. administration of

500 mg kg$^{-1}$. Sample pretreatment and HPLC procedures were described previously (De Bruijn *et al.*, 1985). Blood was collected by heart puncture and two mice were used for the following time points; 1, 5, 15, 30, 45, 60 and 90 min. Mean plasma concentrations of 5′d-FUR were calculated. Area under curve (AUC) and terminal half-life ($t\frac{1}{2},z$) were determined by the trapezoidal rule and linear regression analysis, respectively. Clearance (CL) was determined according to CL = dose/AUC. For comparison between concentrations the Kruskal–Wallis test was used.

### Results

### *Toxicity*

The maximum tolerated dose (MTD) for 5-FU was 50 mg kg$^{-1}$ in nude mice, and 100 mg kg$^{-1}$ for Balb/c and C57Bl/6 mice. No toxic deaths were observed. Since mice tolerated 5′d-FUR at a dose which was 8–10 times higher than 5-FU (Bollag & Hartmann, 1980), we initially used 400 mg 5′d-FUR kg$^{-1}$ in nude mice and 1,000 mg kg$^{-1}$ in Balb/c and C57Bl/6 mice. However, the dose of 5′d-FUR could be increased 20 times to 1,000 and 2,000 mg kg$^{-1}$ in nude mice and normal mice, respectively, which appeared to be the MTD. The moderate weight loss (5–10%) was comparable to that of 5-FU, and was observed only at the first two of the four courses (Figure 2). In non-tumour-bearing mice, the weight of the mice returned to initial values and was followed by normal weight increase. In the tumour bearing Balb/c mice weight loss was observed after discontinuation of the treatment, due to cachexia caused by the tumour. No weight loss was observed in tumour bearing Balb/c and C57Bl/6 mice with 5′d-FUR at doses $\leqslant$1,000 mg kg$^{-1}$. At a dose of 2,500 mg kg$^{-1}$ three of six Balb/c mice died. In nude mice the low dose of 400 mg kg$^{-1}$ caused no weight loss.

### *Antitumour activity*

HNX-DU was resistant to 5-FU at 50 mg kg$^{-1}$ and to 5′d-FUR at 400 and 1,000 mg kg$^{-1}$ (Table II). HNX-KE showed a slight response to 50 mg 5-FU kg$^{-1}$ and at 400 mg 5′d-FUR kg$^{-1}$, but a significant antitumor activity at 1,000 mg 5′d-FUR kg$^{-1}$ (Figure 3). The HNX-G and HNX-E both showed a slight comparable response to 50 mg 5-FU kg$^{-1}$ and 400 mg 5′d-FUR kg$^{-1}$ (Table II). In HNX-E a better antitumour activity was observed at 1,000 mg 5′d-FUR kg$^{-1}$. Higher doses of 5′d-FUR could not be tested in the HNX-G tumour line due to a lack of growth of this line.

Both murine colon tumour lines were studied at various doses of 5′d-FUR. Colon 38 was very sensitive to 5-FU (Figure 4), with several complete responders at 100 mg kg$^{-1}$. Colon 38 showed a similar sensitivity to 5′d-FUR at 1,000 mg kg$^{-1}$; also at lower doses a good response was observed (Table III). Colon 26 was initially tested at 800 and 1,000 mg kg$^{-1}$. Tumour-growth delay was comparable to 5-FU at 100 mg kg$^{-1}$. However, higher doses of 5′d-FUR could be used without lethal toxicity. At 1,500 and 2,000 mg kg$^{-1}$ antitumour activity was significantly enhanced compared to the schedules with the lower doses. GDF was comparable to values which were usually only observed with the sensitive Colon 38 (Table III).

### *Enzyme activities*

A relatively high $K_m$ was observed for 5′d-FUR in all tumours varying from 0.6 to 2 mM (Figure 5), while the maximal activity with 5′d-FUR varied considerably (Table IV). The highest activity in Colon 26 was about 50 times higher than in the tumours with the lowest activity, HNX-DU and HNX-KE. Uridine phosphorylase, measured at saturating uridine concentrations (Table IV), had the highest activity in Colon 26 and the lowest in HNX-KE. Results were expressed per mg protein, the relative differences based on wet weight were also comparable. For comparison the protein content of the tumours is given (Table IV).

Pyrimidine nucleoside phosphorylase with 5-FU as substrate in the reverse direction (Figure 6) was higher with dRib-1-P as co-substrate than with Rib-1-P in all tumours; in the HNX tumours at least 10-fold, but in both colon tumours only 3-fold (Figure 6). With Rib-1-P the highest activity was found in Colon 26 and the lowest in HNX-KE and Colon 38. Among the HNX tumours the highest activity was observed in HNX-E. With dRib-1-P the pattern was different, although the highest activity was still present in Colon 26 and the lowest in Colon 38. However, the activities in the HNX tumours were all higher than in Colon 38 and the difference of HNX-E with Colon 26 was less than with Rib-1-P as co-substrate (Figure 6).

Conversion of 5-FU to either FUMP or FdUMP via FUR or FdUR, respectively, was measured by supplying ATP at a physiological concentration in the assay for pyrimidine nucleoside phosphorylase. Under these conditions an

**Figure 2** Weight loss of mice after treatment with 5′d-FUR (●, control mice; ■, treated mice). Values represent means ±s.e. and are of 5–7 mice. Body weight loss in tumour-bearing animals was corrected for the weight of the tumour. Chemotherapy was started at day 0 and is indicated by arrows. Weight loss of nude mice bearing the other HNX tumour lines was comparable or less, (**a**) HNX-KE tumour-bearing nude mice treated with 1,000 mg kg$^{-1}$; (**b**) Colon 26 tumour-bearing Balb-c treated with 2,000 mg kg$^{-1}$; (**c**) non-tumour-bearing Balb-c mice treated with 2,000 mg kg$^{-1}$.

**Table II** Summary of therapeutic data for the human head and neck tumour lines

| | Treatment | | | | |
|---|---|---|---|---|---|
| *Tumour* | *Drug* | *Dose ($mg\,kg^{-1}$)* | *Schedule* | *Weight loss (%)* | *GDF* |
| HNX-DU | 5-FU | 50 | q7d×2 | 7.6 | 0.4 |
| | 5'd-FUR | 400 | q7d×2 | none | −0.1 |
| | 5'd-FUR | 1,000 | q7d×2 | 3.6 | 0.4 |
| HNX-KE | 5-FU | 50 | q7d×2 | 3.2 | 0.8[a] |
| | 5'd-FUR | 400 | q7d×2 | none | 0.6[b] |
| | 5'd-FUR | 1,000 | q7d×2 | 4.2 | 2.1[b] |
| HNX-G | 5-FU | 50 | q7d×2 | 6.4 | 1.0[a] |
| | 5'd-FUR | 400 | q7d×2 | 3.5 | 0.6 |
| HNX-E | 5-FU | 50 | q7d×2 | 3.8 | 0.8[b] |
| | 5'd-FUR | 400 | q7d×2 | none | 0.9[b] |
| | 5'd-FUR | 1,000 | q7d×2 | <5 | 1.3[b] |

Significantly different from untreated animals at the levels: [a]$0.001<P<0.01$; [b]$0.01<P<0.05$. No regressions were observed.

**Figure 3** Antitumour activity of 5-FU and 5'd-FUR in HNX-KE. Values represent means ±s.e. of 5–10 tumours. Chemotherapy is indicated by the arrows. Relative tumour volumes were plotted until tumour size was 4 times its initial volume at the start of treatment. (**a**) ○, control; ■, 50 mg 5-FU $kg^{-1}$; ●, 400 mg 5'd-FUR $kg^{-1}$; (**b**) ○, control; ◆, 1,000 mg 5'd-FUR $kg^{-1}$.

estimate of the conversion of 5-FU to the nucleotide will be obtained (Peters et al., 1986a). This conversion was termed 'chanelling', although the enzymes responsible for these conversions do not exist in an enzyme complex. The concentration of FUR in the reacton mixture exceeded the $K_m$ values for phosphorylation of FUR (Greenberg et al., 1977), so the concentration of FUR was not rate-limiting. The rate of chanelling was measured in the presence of a phosphatase inhibitor to prevent degradation of newly formed nucleotide. With Rib-1-P as co-substrate the presence of this inhibitor resulted in a higher amount of FUMP (about 1.5-fold) in HNX-KE and HNX-G and in Colon 38; with dRib-1-P a higher amount of FdUMP was found in HNX-KE, HNX-G and Colon 26 (about 1.4-fold) and in Colon 38 (2.7-fold).

Chanelling of 5-FU to FUMP was highest in Colon 26 and very low in Colon 38 (Figure 7). All HNX tumours showed an at least 10 times higher rate than Colon 38, but also less than 5% of that in Colon 26. Chanelling of 5-FU to FdUMP showed a completely different pattern. The activity was highest in Colon 26 but the difference with the other tumours was less, the activity in HNX-DU and KE was 50% of that in Colon 26. The rate of chanelling in the other three tumour lines was much lower.

Direct conversion of 5-FU catalysed by OPRT with PRPP as the essential co-substrate was measured in the presence of a nucleotidase inhibitor; in all tumour lines (except Colon 38 and HNX-KE) this resulted in a higher activity (1.5–2.0-fold) than in the absence. The highest activity of OPRT was observed in Colon 26 and Colon 38 (Figure 8); from the HNX tumours the activity was highest in HNX-KE.

*Phamacokinetics*

Differences between plasma levels of 5'd-FUR and 5-FU in mouse strains following i.p. injection of 500 mg $kg^{-1}$ were not significant ($P>0.05$). Table V shows data of the AUC, $t_{\frac{1}{2},z}$ and CL for nude mice and normal mice. The data of nude mice are comparable with those of normal mice and are not suggestive of strain differences as found between WAG/Rij and Wistar rats (De Bruijn et al., submitted for publication). 5-FU levels derived from 5'd-FUR were comparable in both mouse strains, as well as plasma concentrations of the first breakdown product of 5-FU 5-fluorodihydrouracil (data not shown).

### Discussion

Patients with inoperable head and neck cancer have only limited benefit from chemotherapy treatment. Initial responses are often seen, as with the cisplatin/5-FU schedule, but enhancement of survival is minimal (Tannock & Browman, 1986). There is still a need for active drugs for this type of cancer. An attractive way to select agents for phase II trials is the use of human xenograft tumour lines (Braakhuis et al., 1983; Winograd et al., 1987). The present study demonstrated that for human xenografts resistant to 5-FU the therapeutic efficacy might be increased by treatment with the 5-FU produg 5'd-FUR. Also the sensitivity of the murine colon tumour Colon 26 could be enhanced. The sensitivity of the 5-FU sensitive Colon 38 was not affected. Mice tolerated a dose of 5'd-FUR 20 times higher than that for 5-FU. In addition to this relatively low tissue toxicity the improved therapeutic efficacy might also be related to the rate of activation of 5'd-FUR to 5-FU. In Colon 26 the activity of pyrimidine nucleoside phosphorylase with 5'd-FUR is up to 15 times higher than in the other tumours. Furthermore, all sensitive (GDF>2) tumours have a relatively high activity of OPRT, which might play a key role in the further activation of 5-FU.

The sensitivity of tumours to 5'd-FUR might be correlated with pyrimidine nucleoside phosphorylase (Ishitsuka et al., 1980; Armstrong et al., 1980, 1983a; Hartmann & Matter, 1982) although no strict correlation has been demonstrated (Hartmann & Matter, 1982; Peters et al., 1986a). This discrepancy might be related to the role of uridine phosphorylase in the activation of 5'd-FUR. Uridine phosphorylase might be responsible for conversion of 5'd-

**Figure 4** Antitumour activity of 5-FU and 5′d-FUR in Colon 26 and Colon 38. Values represent means ±s.e. of 8–12 tumours. In Colon 26 chemotherapy had to be discontinued due to cachexia caused by the tumours. The relative tumour volumes were plotted until the median day of death (values are of at least 4 mice). For Colon 38 tumour volumes were plotted until they reached 2,000 mm$^3$. (**a**) Colon 26: ○, control; ● 1,500 mg 5′d-FUR kg$^{-1}$; ■, 2,000 mg 5′d-FUR kg$^{-1}$. (**b**) Colon 38: ○, control; ■, 100 mg FU kg$^{-1}$; ●, 1,000 mg 5′d-FUR kg$^{-1}$.

**Table III** Summary of therapeutic data for the colon tumours

| | *Treatment* | | | | | |
|---|---|---|---|---|---|---|
| *Tumour* | *Drug* | *Dose (mg kg$^{-1}$)* | *Schedule* | *Weight loss (%)* | *ILS (C26) CR (38)* | *GDF* |
| Colon 26 | 5-FU | 100 | q7d × 2 | 5.2 | 161 | 1.5[a] |
| | 5-FU | 250 | q7d × 1 | 12.1 | (toxic death) | |
| | 5′d-FUR | 800 | q7d × 3 | 4.3 | 300 | 1.7[a] |
| | 5′d-FUR | 1,000 | q7d × 3 | 6.2 | 257 | 3.9[a] |
| | 5′d-FUR | 1,500 | q7d × 4 | 6.8 | 333 | 4.5[a] |
| | 5′d-FUR | 2,000 | q7d × 4 | 8.0 | 356 | 6.6[a] |
| Colon 38 | 5-FU | 60 | q7d × 4 | none | none | 2.4[b] |
| | 5-FU | 100 | q7d × 4 | 2.4 | 4/18 | 5.2[a] |
| | 5′d-FUR | 600 | q7d × 4 | none | 6/18 | 4.8[a] |
| | 5′d-FUR | 800 | q7d × 4 | none | 6/18 | 4.4[a] |
| | 5′d-FUR | 1,000 | q7d × 4 | no | 2/9 | 5.5[a] |

ILS: increase in life-span, calculated as T/C% = (median life span treated mice)/(median life span untreated mice) × 100%; the first day of treatment was used as day 0. Only for Colon 26 is the ILS given, since C57Bl/6 mice bearing Colon 38 were killed when tumour volume exceeded 2,000 mm$^3$. For Colon 38 the number of complete responders (CR) is given, the GDF is calculated from the remaining tumours. Significantly different from untreated animals at the level: [a]$P < 0.001$; [b]$0.001 < P < 0.01$.

FUR to 5-FU (Ishitsuka *et al.*, 1980) but 5′d-FUR is also a substrate for thymidine phosphorylase (Siegel & Lin, 1986). In our studies it appeared that the rate of phosphorolysis of 5′d-FUR did not correlate with that of uridine, and the pattern of activity was completely different for both substrates. Actually the activity of pyrimidine nucleoside phosphorylase with 5′d-FUR correlated with that of 5-FU and dRib-1-P as substrates. This conversion of 5-FU to FUdR and the cleavage of FUdR are mainly catalysed by a thymidine and/or a uridine-deoxyuridine phosphorylase (Woodman *et al.*, 1980; Peters *et al.*, 1986*a*). Enzyme kinetics of pyrimidine nucleoside phosphorylase with 5′d-FUR as a substrate were comparable to other studies, about 1 mM (Armstrong & Diasio, 1980; Choong & Lee, 1986; Miwa *et al.*, 1981), which is much higher than that for uridine (Leyva *et al.*, 1983; Laurensse *et al.*, 1988) or thymidine (Wataya & Santi, 1981). So, 5′d-FUR has a low affinity for pyrimidine phosphorylases and might be a substrate for various phosphorylases (Woodman *et al.*, 1980; Choong & Lee, 1986).

Resistance of the HNX lines to 5-FU might be related to several factors, such as a deficient activation of 5-FU, differences in the inhibition of the target enzyme thymidylate synthase, high intratumoral levels of thymidine or deoxyuridine or the incorporation of 5-FU into RNA (Pinedo & Peters, 1988). However, the mechanism of 5′d-FUR cytotoxicity is directly analogous to that for 5-FU, inhibition of thymidylate synthase being the most potent mechanism (Armstrong *et al.*, 1983*b*). Thus differences between effects of 5-FU and 5′d-FUR are most likely related to activation of 5′d-FUR to 5-FU and subsequently to active nucleotides.

In this panel of tumours uridine phosphorylase appears not be be related to the sensitivity of the tumours to 5′d-

**Figure 5** Lineweaver–Burk plots for determination of the $K_m$ for 5′d-FUR in the various tumour lines. Før each line a representative experiment is shown out of 3–4. v, nmol $h^{-1}$ mg $protein^{-1}$.

**Table IV** Activities of pyrimidine nucleoside phosphorylase with 5′d-FUR and uridine as substrates

| *Tumour line* | *5′d-FUR* $V_{max}$ | *5′d-FUR* $K_m$ | *Uridine activity* | *mg protein per mg wet weight* |
|---|---|---|---|---|
| HNX-E | 2,300+460 | 1.16±0.30 | 179±68 | 24.9±2.5 |
| HNX-DU | 273±28 | 0.80±0.11 | 124±14 | 27.8±6.9 |
| HNX-KE | 366±54 | 1.16±0.09 | 73±6 | 36.6±2.8 |
| HNX-G | 2,565±293 | 1.02±0.34 | 94±7 | 22.8±2.7 |
| Colon 26 | 9.892±2,097 | 1.89±0.54 | 9,040±1,525 | 53.8±9.0 |
| Colon 38 | 671±238 | 2.19±1.56 | 344±65 | 47.3±11.1 |

Enzyme activities are given as nmol of 5-FU or uracil formed per h per mg protein, $K_m$ is given in mM. $V_{max}$ and $K_m$ values were determined in separate tumours. Values are means ±s.e. of 3–5 different tumours. For comparison the protein content of the tumours is given; protein was determined in the 11,000*g* supernatant using the Biorad dye exclusion assay.

**Figure 6** Activity of pyrimidine nucleoside phosphorylase with 5-FU as substrate and Rib-1-P (filled columns) or dRib-1-P (hatched columns) as co-substrate. Values represent means ±s.e. of 3–5 different tumours.

**Figure 7** Synthesis of FUMP and FdUMP from 5-FU via FUR or FUdR, respectively. The 'channeling' reaction was measured with Rib-1-P (filled columns) or dRib-1-P (hatched columns) as co-substrates in the presence of ATP. Values represent means ±s.e. of 3–5 different tumours.

FUR. The very sensitive Colon 38 and Colon 26 have a very low and high rate of 5′d-FUR phosphorolysis, respectively. However, the sensitive HNX-KE has a lower activity than HNX-E and HNX-G. So, other factors than the rate of phosphorolysis of 5′d-FUR play an important role in the sensitivity to 5′d-FUR, such as a subsequent

**Figure 8** Activity of OPRT with 5-FU as substrate and PRPP as co-substrate. Values represent means ±s.e. of 3–5 different tumours.

**Table V** Pharmacokinetic parameters of 5′d-FUR and 5-FU in normal and nude mice

| *Parameter* | *Compound* | *Normal mice* | *Nude mice* |
|---|---|---|---|
| AUC | 5′d-FUR | 110 | 130 |
| ($\mu$mol min ml$^{-1}$) | 5-FU | 6.1 | 6.4 |
| $t\frac{1}{2}, z$ (min) | 5′d-FUR | 30.0 | 35.0 |
| | 5-FU | 29.0 | 24.2 |
| CL | 5′d-FUR | 18.4 | 15.7 |
| (ml min$^{-1}$ kg$^{-1}$) | | | |

Mice received an i.p. injection of 500 mg 5′d-FUR kg$^{-1}$. Mean plasma concentration–time curves from $t=1$ to $t=90$ min were calculated for two mice per time-point. AUC, $t\frac{1}{2}, z$ and CL were determined by model-independent methods.

phosphorylation to nucleotides and incorporation into RNA (Armstrong *et al.*, 1983*a*). Cory & Carter (1982) demonstrated that 5-FU and 5′d-FUR are not activated in the same way in order to show growth-inhibition. Activation to FdUMP via FUdR is unlikely because of low endogenous levels of the co-substrate dRib-1-P. Levels of Rib-1-P are usually high enough for activation of 5-FU to FUMP via FUR, but this pathway was low in the 5′d-FUR sensitive Colon 38, high in the 5′d-FUR resistant HNX-DU, and very high in Colon 26, and might only be important for Colon 26. However, this way of phosphorylation of 5-FU formed from 5′d-FUR is unlikely (Peters *et al.*, 1986*a*), which could be partially due to: (a) the presence of the modified pentose phosphate 5-deoxy-ribose-1-phosphate which might compete with the substrate Rib-1-P and (b) an interference of 5′d-FUR itself with pyrimidine nucleoside phosphorylase. The only alternative for phosphorylation of 5-FU will be direct conversion to FUMP. The substrate for this reaction, PRPP, could be decreased by 5′d-FUR only in WiDr cells, the most sensitive cell line in this panel (Peters *et al.*, 1985, 1986*a*). WiDr cells had a relatively high activity of OPRT both with the analogue substrate 5-FU and the natural substrate orotic acid (Peters *et al.*, 1985, 1986*a*). In the present panel the most sensitive lines Colon 26, Colon 38 and HNX-KE have a relatively high activity of OPRT with 5-FU. So, only tumours with a sufficient capacity to convert 5′d-FUR to 5-FU and a subsequent effective conversion of 5-FU to nucleotides may be sensitive to 5′d-FUR.

Differences between *in vivo* drug handling of different mouse strains have not been observed and therefore extra-tumoral exposure is considered to be comparable. Thus, differences between 5′d-FUR effects on tumours in nude and normal mice cannot be explained by differences in extra-tumoral drug handling.

5-FU resistant tumours may not use efficiently the pathway catalysed by OPRT for 5-FU activation, possibly due to a low availability of PRPP or a high activity of pyrimidine nucleoside phosphorylase. Thus inhibition of pyrimidine nucleoside phosphorylase may lead to a more efficient use of the OPRT pathway, and therapy of 5-FU resistant tumours, such as several head and neck and colon cancers, might be improved by an efficient use of the OPRT pathway. Biochemical modulators, such as inhibitors of uridine phosphorylase may prove to be potential chemotherapeutic agents (Siegel & Lin, 1986).

Several human colon tumours have a relatively high activity of OPRT compared to adjacent normal mucosal tissue (Peters *et al.*, 1986*b*). So, colorectal cancer might be an attractive tumour type to be treated with 5′d-FUR, and recent results showed an improved therapeutic effect in rectosigmoid colorectal cancer (Abele *et al.*, 1988). An advantage over 5-FU might be expected since the 5-FU sensitive tumours will also respond to 5′d-FUR while some 5-FU resistant or relatively resistant tumours might show a better response. Other schedules or ways of administration might be explored to circumvent the observed neurotoxicity.

In conclusion, a better therapeutic efficacy was achieved with 5′d-FUR for several lines than with 5-FU. The enhanced sensitivity to 5′d-FUR might not strictly be related to its rate of conversion to 5-FU. Although a certain amount of activity is essential to convert 5′d-FUR to 5-FU, further metabolism to nucleotides might be limiting. This might lead to an improved therapeutic efficacy of 5′d-FUR compared to 5-FU in tumours with a high OPRT activity such as HNX-KE and Colon 26. These findings indicate that further studies aiming at enhancing of therapeutic efficacy observed with 5′d-FUR are warranted.

This work was supported by the Netherlands Cancer Foundation (Koningin Wilhelmina Fonds) by grants IKA 83-16 and AUKC-VU 82-11, and by Hoffmann-La Roche, Mijdrecht, The Netherlands. We also thank Dr J. van Dijk, Mrs M. Bagnay and Mr E.J. Schoevers for their contributions. Dr G.J. Peters is a senior research fellow of the Royal Netherlands Academy of Sciences.

### References

ABELE, R., KAPLAN, E., GROSSENBACHER, R., SCHMIDT, H.J. & CAVALLI, F. (1984). Phase II study of doxifluridine in advanced squamous cell carcinoma of the head and neck. *Eur. J. Cancer Clin. Oncol.*, **20**, 333.

ALBERTO, P., MERMILLOD, B., GERMANO, G. & 6 others (1988). A randomized comparison of Doxifluridine and fluorouracil in colorectal carcinoma. *Eur. J. Cancer Clin. Oncol.*, **24**, 559.

ARMSTRONG, R.D. & DIASIO, R.B. (1980). Metabolism and biological activity of 5′-deoxy-5-fluorouridine. *Cancer Res.*, **40**, 3333.

ARMSTRONG, R.D. & DIASIO, R.B. (1981). Selective activation of 5′-deoxy-5-fluorouridine by tumor cells as a basis for an improved therapeutic index. *Cancer Res.*, **41**, 4891.

ARMSTRONG, R.D., GESMONDE, J., WU, T. & CADMAN, E. (1983*a*). Cytotoxic activity of 5′-deoxy-5-fluorouridine in cultured human tumors. *Cancer Treat. Rep.*, **67**, 541.

ARMSTRONG, R.D., CONNOLLY, K.M., KAPLAN, A.M. & CADMAN, E. (1983*b*). Mechanism of cytotoxic activity of 5′-deoxy-5-fluorouridine. *Cancer Chemother. Pharmacol.*, **11**, 102.

BARANKIEWICZ, J. & HENDERSON, J.F. (1977). Determination of ribose 1-phosphate in ascites cells. *Biochem. Med.*, **17**, 45.

BOLLAG, W. & HARTMANN, H.R. (1980). Tumor growth inhibitory effects of a new fluorouracil derivative: 5′-deoxy-5-fluorouridine. *Eur. J. Cancer*, **16**, 427.

BRAAKHUIS, B.J.M., SCHOEVERS, E.J., HEINERMAN, E.C.M., SNEEUWLOPER, G. & SNOW, G.B. (1983). Chemotherapy of human head and neck cancer xenografts with three clinically active drugs: *cis*-platinum, bleomycin and methotrexate, *Br. J. Cancer*, **48**, 711.

BRAAKHUIS, B.J.M., SNEEUWLOPER, G. & SNOW, G.B. (1984). The potential of the nude mouse xenograft model for the study of head and neck cancer. *Arch. Otorhinolaryngol.*, **239**, 69.

BRAAKHUIS, B.J.M., LEYVA, A., SCHOEVERS, E.J., BOERRIGTER, G.H., SCHORNAGEL, J.H. & SNOW, G.B. (1985). Lack of effect of methotrexate on human head and neck tumours transplanted in aythmic, nude mice. *Acta Otolaryngol.*, **99**, 208.

BRAAKHUIS, B.J.M., LEYVA. A., PINEDO, H.M. & SNOW, G.B. (1986). Antitumour effect of 5-aza-2′deoxycytidine in human head and neck cancer xenografts. *Proc. Am. Assoc. Cancer Res.*, **27**, 299.

BRAAKHUIS, B.J.M. & SNOW, G.B. (1988). *Activity of Conventional Drugs in Head and Neck Cancer Xenografts*. ESO Monographs, Human Xenografts in Anticancer Drug Development, p. 37. Springer Verlag: Heidelberg.

CHOONG, Y.S. & LEE, S.P. (1985). The degradation of 5′-deoxy-5-fluorouridine by pyrimidine nucleoside phosphorylase in normal and cancer tissues. *Clin. Chim. Acta*, **149**, 175.

CORY, J.G. & CARTER, G.L. (1982). Evidence that 5′-deoxy-5-fluorouridine may not be activated by the same mechanism as 5-fluorouracil. *Biochem. Pharmacol.*, **31**, 2841.

DE BRUIJN, E.A., VAN OOSTEROM, A.T., TJADEN, U.R., VAN REEUWIJK, H.J.E.M. & PINEDO, H.M. (1985). Pharmacology of 5′-deoxy-5-fluorouridine in patients with resistant ovarian cancer. *Cancer Res.*, **45**, 5931.

GREENBERG, N., SCHUMM, D.E. & WEBB, T.E. (1977). Uridine kinase activities and pyrimidine nucleoside phosphorylation in fluoropyrimidine-sensitive and resistance cell lines of the Novikoff hepatoma. *Biochem. J.*, **164**, 379.

HARTMANN, H.R. & MATTER, A. (1982). Antiproliferative action of a novel fluorinated uridine analog, 5′-deoxy-5-fluorouridine, measured *in vitro* and *in vivo* on four different murine cell lines. *Cancer Res.*, **42**, 2412.

HURTELOUP, P., ARMAND, J.P., CAPPELAERE, P. & 16 others (1986). Phase II Clinical evaluation of doxifluridine. *Cancer Treat. Rep.*, **70**, 731.

ISHITSUKA, H., MIWA, M., TAKEMOTO, K., FUKUOKA, K., ITOGA, A. & MARUYAMA, H.B. (1980). Role of uridine phosphorylase for antitumor activity of 5′-deoxy-5-fluorouridine. *Gann*, **71**, 112.

KOK, R.M., DE JONG, A.P.J.M., VAN GROENINGEN, C.J., PETERS, G.J. & LANKELMA, J. (1984). Highly sensitive determination of 5-fluorouracil in human plasma by capillary gas chromatography and negative ion chemical ionization mass spectrometry. *J. Chromatogr.*, **343**, 59.

LAURENSSE, E., PINEDO, H.M. & PETERS, G.J. (1988). A sensitive non-radioactive assay for pyrimidine nucleoside phosphorylase using reversed phase high-performance chromatography. *Clin. Chim. Acta*, **178**, 71.

LEYVA, A., KRAAL, I., LANKELMA, J., DELEMARRE, J.F.M. & PINEDO, H.M. (1983). High uridine phosphorylase activity in human melanoma. *Anticancer Res.*, **3**, 227.

MIWA, M., NAKAMURA, J. & ISHITSUKA, H. (1981). A simple and convenient assay method for phosphorolysis of 5′-deoxy-5-fluorouridine. *Gann*, **72**, 965.

PETERS, G.J., LAURENSSE, E., LANKELMA, J., LEYVA, A. & PINEDO, H.M. (1984). Separation of several 5-fluorouracil metabolites in various melanoma cell lines. Evidence for the synthesis of 5-fluorouracil-nucleotide sugars. *Eur. J. Cancer Clin. Oncol.*, **20**, 1425.

PETERS, G.J., LAURENSSE, E., LEYVA, A. & PINEDO, H.M. (1985). The concentration of 5-phosphoribosyl-1-pyrophosphate in monolayer cells and the effect of various pyrimidine anti-metabolites. *Int. J. Biochem.*, **17**, 95.

PETERS, G.J., LAURENSSE, E., LEYVA, A., LANKELMA, J. & PINEDO, H.M. (1986*a*). Sensitivity of human, murine and rat cells to 5-fluorouracil and 5′deoxy-5-fluorouridine in relation to drug-metabolizing enzymes. *Cancer Res.*, **46**, 20.

PETERS, G.J., LAURENSSE, E., LEYVA, A. & PINEDO, H.M. (1986*b*). Tissue homogenization using a microdismembrator for the measurement of enzyme activities. *Clin. Chim. Acta*, **158**, 193.

PETERS, G.J., LAURENSSE, E., LEYVA, A. & PINEDO, H.M. (1987*a*). Purine nucleosides as cell-specific modulators of 5-fluorouracil metabolism and cytotoxity. *Eur. J. Cancer Clin. Oncol.*, **23**, 1869.

PETERS, G.J., VAN DIJK, J., NADAL, J.C., VAN GROENINGEN, C.J., LANKELMA, J. & PINEDO, H.M. (1987*b*). Diurnal variation in the therapeutic efficacy of 5-fluorouracil against murine colon cancer. *In Vivo*, **1**, 113.

PINEDO, H.M. & PETERS, G.J. (1988), 5-Fluorouracil: Biochemistry and pharmacology. *J. Clin. Oncol.*, **6**, 1653.

SIEGEL, S.A. & LIN, T.-S. (1986). Inhibitors of uridine phosphorylase: Potential chemotherapeutic agents. *Drugs Future*, **11**, 961.

SOMMADOSSI, J.-P., AUBERT, C., CANO, J.-P., GOUVEIA, J., RIBAUD, P. & MATHE, G. (1983). Kinetics and metabolism of a new fluoropyrimidine, 5′-deoxy-5-fluorouridine in humans. *Cancer Res.*, **43**, 930.

TANNOCK, I.F. & BROWMAN, G. (1986). Lack of evidence for a role of chemotherapy in the routine management of locally advanced head and neck cancer. *J. Clin. Oncol.*, **4**, 1121.

WATAYA, Y., SANTI, D.V. (1981). Continuous spectrophotometric assay of thymidine phosphorylase using 5-nitro-2′-deoxyuridine as substrate. *Anal. Biochem.*, **112**, 96.

WINOGRAD, B., BOVEN, E., LOBBEZOO, M.W. & PINEDO, H.M. (1987). Human xenografts in the nude mice and their value as test models in anticancer drug development. *In Vivo*, **1**, 1.

WOODMAN, P.W., SARIL, A.M. & HEIDELBERGER, C. (1980). Specificity of pyrimidine nucleoside phosphorylases and the phosphorylysis of 5-fluoro-2′-deoxyuridine. *Cancer Res.*, **40**, 507.